# Molecular Identification and Survey of Trichomonad Species in Pigs in Shanxi Province, North China

**DOI:** 10.3390/vetsci11050203

**Published:** 2024-05-07

**Authors:** Zi-Rui Wang, Qing-Xin Fan, Jin-Long Wang, Shuo Zhang, Yu-Xuan Wang, Ze-Dong Zhang, Wen-Wei Gao, Xing-Quan Zhu, Qing Liu

**Affiliations:** 1 Laboratory of Parasitic Diseases, College of Veterinary Medicine, Shanxi Agricultural University, Jinzhong 030801, China; wangzirui8091@126.com (Z.-R.W.); fanqingxin2000@126.com (Q.-X.F.); jinlong1279@163.com (J.-L.W.); shuoshuozhang0828@163.com (S.Z.); wangyuxuan1113@126.com (Y.-X.W.); zzd18203541696@163.com (Z.-D.Z.); sxndgaowenwei@163.com (W.-W.G.); xingquanzhu1@hotmail.com (X.-Q.Z.); 2 Key Laboratory of Veterinary Public Health of Higher Education of Yunnan Province, College of Veterinary Medicine, Yunnan Agricultural University, Kunming 650201, China

**Keywords:** trichomonads, pigs, prevalence, sequence analysis, Shanxi Province

## Abstract

**Simple Summary:**

Three trichomonad species (*Tritrichomonas foetus*, *Tetratrichomonas buttreyi*, and *Pentatrichomonas hominis*) may affect pig production. Moreover, *P. hominis* is a zoonotic species. In the current investigation, 362 fecal samples collected from pigs in three representative counties in Shanxi Province, North China, were molecularly tested for the three trichomonad species. The results showed that *T. buttreyi* was detected as the most common species, with a prevalence of 49.72%. Its prevalence was associated with region and age. Only one sample was tested positive for *T. foetus*, and this isolate showed minor allelic variations compared with those reported previously. For *T. buttreyi*, eight distinct sequence types were obtained, and only two displayed 100% nucleotide homology with the corresponding sequences reported previously. For the first time, we reported the prevalence and genetic characterization of *T. foetus* and *T. buttreyi* in pigs in Shanxi Province, North China, which provides baseline information for planning control strategies.

**Abstract:**

Several trichomonad species have already been identified in pigs, and their pathogenic potential may not be ruled out. To date, however, no information is available regarding the prevalence of trichomonads in pigs in Shanxi Province, North China. In the present study, a total of 362 fecal samples collected from pigs in three representative counties (Qi, Jishan, and Shanyin) in this province were examined for *Tetratrichomonas buttreyi*, *Tritrichomonas foetus*, and *Pentatrichomonas hominis* using a nested polymerase chain reaction (PCR) with primers targeting the small subunit ribosomal RNA (SSU rRNA) gene. The overall prevalence of *T. buttreyi* was 49.72%, and region and age were found to be significantly associated with *T. buttreyi* infection, respectively. Only one pig fecal sample from Qi County was found to be positive for *T. foetus*, and all samples were negative for *P. hominis*. Molecular evolutionary analysis revealed that some *T. buttreyi* isolates showed complete genetic identity with those reported previously, and some *T. buttreyi* isolates and one *T. foetus* isolate showed minor allelic variations compared with those reported previously. This is the report of the molecular epidemiology of *T. foetus* and *T. buttreyi* in pigs in Shanxi Province, North China. These findings not only enrich the knowledge on the distribution of these trichomonad species in pigs in China but also provide baseline information for planning future research and control strategies.

## 1. Introduction

Trichomonads are amitochondriate flagellated protists that belong to the order Trichomonadida within the phylum Parabasalia [1]. Several trichomonad species have been found to inhabit the intestinal tracts of pigs [2,3], of which *Tritrichomonas foetus* has been found to be associated with the reproductive tract disease of cattle [3,4]. In addition, *T. foetus* has been confirmed to be responsible for large bowel diarrhea in cats [5,6]. In general, pigs naturally infected with *T. foetus* have no apparent clinical signs [3]. However, this trichomonad species was reported to cause lung infections in a 40-day-old piglet [7].

*Tetratrichomonas buttreyi*, originally found in pigs, was reported to be commensal to both cattle and pigs [8]. However, the potential pathogenicity of *T. buttreyi* is possible, because a previous report indicated that the excessive multiplication of trichomonads could cause pathogenic effects [9].

*Pentatrichomonas hominis* is a trichomonad species reported in humans and important economic animals including pigs [10,11,12]. Though *P. hominis* was originally thought to be a commensal organism, it has recently been detected in the feces of cats and dogs with diarrhea [13,14]. Hence, the pathogenic potential of *P. hominis* in pigs may not be ruled out. Moreover, a previous study reported that *P. hominis* may be strongly linked with human gastrointestinal cancers [15].

Knowledge regarding the prevalence of the three trichomonad species mentioned above (*T. foetus*, *T. buttreyi*, and *P. hominis*) is of great importance from both human and veterinary medicine standpoints. To date, the prevalence of *T. foetus*, *T. buttreyi*, and *P. hominis* in pigs in two Chinese provinces has been investigated [10,16]. The pig industry plays an important role in animal husbandry in Shanxi Province, North China. However, no information is currently available regarding the infection status of the three trichomonad species in pigs in this province. Thus, the prevalence of *T. foetus*, *T. buttreyi*, and *P. hominis* in pigs in Shanxi Province, North China was determined in the present study, followed by sequencing and phylogenetic analysis.

## 2. Materials and Methods

### 2.1. Sample Collection and DNA Extraction

In November 2020, a total of 362 fresh fecal samples were collected from pigs in the following counties: Shanyin (*n* = 183), Qi (*n* = 68), and Jishan (*n* = 111). The sampling locations included the northern, central, and southern parts of Shanxi Province, respectively. Along with fecal sample collection, age information was recorded for each animal from face-to-face interviews with the breeders. The fecal samples were transported immediately to the laboratory using an airtight foam box containing ice packs and then kept in a refrigerator at −20 °C until further use. Extraction of genomic DNA from 0.2 g of each fecal sample was carried out by using the EZNA Stool DNA extraction kit (Omega Bio-tek Inc., Norcross, GA, USA) according to the manufacturer’s specifications, and the obtained genomic DNA samples were kept frozen at −20 °C until further use as templates for polymerase chain reaction (PCR) amplification.

### 2.2. PCR Analysis

The positivity of *T. buttreyi*, *T. foetus* and *P. hominis* was determined by nested PCR amplification of a fragment of the small subunit ribosomal RNA (SSU rRNA) gene, and the first-round PCR was performed using the same primer set (forward: 5′-GCGCCTGAGAGATAGCGACTA-3′; reverse: 5′-GGACCTGTTATTGCTACCCTCTTC-3′) with the following cycling conditions: initial denaturation at 95 °C for 10 min, followed by 30 cycles of 95 °C for 1 min, 60 °C for 1 min, 72 °C for 1 min, and a final extension step of 72 °C for 10 min [10]. To identify *T. foetus*, the first-round PCR product was subjected to a second-round PCR reaction with inner primers (forward: 5′-GGTTGTTTGTATAGGATTGC-3′; reverse: 5′-TGCCCTCATAAAAGGACAA-3′) and cycle conditions as follows: an initial denaturation step at 95 °C for 10 min, followed by 30 cycles of 30 s at 95 °C, 30 s at 54 °C, 30 s at 72 °C, and a final 10 min at 72 °C. To identify *T. buttreyi*, the first-round PCR product was subjected to a second-round PCR reaction with inner primers (forward: 5′-GTTTTTTCTCAGGCAGCAATG-3′; reverse: 5′-GCAACCTAGAAACCTAGGCG-3′) and cycle conditions as follows: an initial denaturation step at 95°C for 10 min, followed by 30 cycles of 45 s at 95 °C, 45 s at 60 °C, 45 s at 72 °C, and a final 10 min at 72 °C. To identify *P. hominis*, the first-round PCR product was subjected to a second-round PCR reaction with inner primers (forward: 5′-TGTAAACGATGCCGACAGAG-3′; reverse: 5′-CAACACTGAAGCCAATGCGAGG-3′) and cycle conditions as follows: an initial denaturation step at 95 °C for 10 min, followed by 30 cycles of 30 s at 95 °C, 30 s at 55 °C, 30 s at 72 °C, and a final 10 min at 72 °C.

The PCR reaction mixture (a final volume of 25 μL) was composed of 2.5 μL 10 × PCR buffer (Mg^2+^ free), 0.2 mM of dNTP mixture, 1.5 mM of MgCl_2_, 0.125 μL of Ex Taq polymerase (5 U/μL) (TaKaRa, Dalian, China), 2 μL of genomic DNA (used as a DNA template in the primary PCR) or the primary PCR product (used as a DNA template in the secondary PCR), and 0.4 μM of each primer. After amplification, the sizes of the secondary PCR products were checked by migration on 1.5% agarose gel electrophoresis.

### 2.3. Sequencing and Phylogenetic Analysis

Positive PCR products were excised from the gels and purified, followed by Sanger sequencing in both directions by Sangon Biotech Co., Ltd. (Shanghai, China) using an Applied Biosystems™ 3730XL DNA Analyzer (Thermo Fisher Scientific, Waltham, MA, USA). The nucleotide sequences obtained in this study were edited and compared for similarity with those available in the archival database GenBank by using the Basic Local Alignment Search Tool (BLAST). A phylogenetic tree was constructed by using the neighbor-joining (NJ) method described previously [16]. Genetic distances were calculated in MEGA 7 software using the Kimura parameter-2 model [17], and the robustness of the findings was evaluated with 1000 bootstrap replicates [18].

### 2.4. Statistical Analysis

Chi-square analysis was performed to assess the relationships between parasitic infections and the different independent factors addressed in the present study using the software SPSS 26.0 (IBM, Chicago, IL, USA). The magnitude of the association was tested by calculating odds ratios (ORs) with 95% confidence intervals (95% CIs). A *p*-value less than 0.05 was considered to indicate a statistically significant result.

## 3. Results

### 3.1. Prevalence of Trichomonads in Pigs

**Figure 1:**
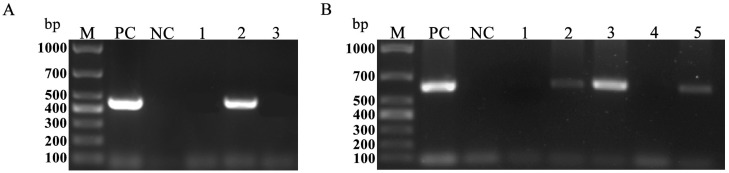
Agarose gel electrophoresis of the PCR products. (**A**) Lane M: 1000 bp DNA ladder; lane PC: positive control; lane NC: negative control; lane 1–3: nested PCR amplification with primers specific for a 452-bp segment of the *T. foetus* SSU rRNA gene using the genomic DNA extracted from each pig fecal sample as a template. (**B**) Lane M: 1000 bp DNA ladder; lane PC: positive control; lane NC: negative control; lane 1–5: nested PCR amplification with primers specific for a 623-bp segment of the *T. buttreyi* SSU rRNA gene using the genomic DNA extracted from each pig fecal sample as a template.

As shown in Figure 1, nested PCR amplification yielded specific bands of the expected sizes (approximately 452 bp and 623 bp, respectively). Of the 362 pig fecal samples examined by nested PCR, 180 (49.72%, 95% CI: 44.57–54.87) were positive for *T. buttreyi* (Table 1). The prevalence of *T. buttreyi* was significantly influenced by region (*p* < 0.001). Among the three counties, the highest prevalence of *T. buttreyi* was observed in Qi (80.88%, 95% CI: 71.54–90.23), followed by Jishan (51.37%, 95% CI: 44.12–58.61) and Shanyin (27.93%, 95% CI: 19.58–36.27) (Table 1). Regarding the age groups, pigs aged > 6 months had the highest prevalence (56.80%, 95% CI: 48.12–65.48), whereas the lowest prevalence was observed in pigs aged ≤4 months (36.73%, 27.19–46.28) (Table 1). A statistically significant difference in its prevalence was observed among different age groups (*p* = 0.008). Only one pig fecal sample from Qi County was tested positive for *T. foetus*, and no pig fecal samples were tested positive for *P. hominis*.

### 3.2. DNA Sequence Analysis

The sequence of the *T. foetus* SSU rRNA gene obtained in the present study has been deposited in GenBank with the accession number: PP256583. Sequence comparisons using BLAST showed that the sequence obtained in the present study had a high degree of sequence similarity (99.78%) with those of *T. foetus* from pigs (MK801504, KX833160, and KM205209), cattle (AY055799), and cats (OR033176 and AF466749).

**Figure 2:**
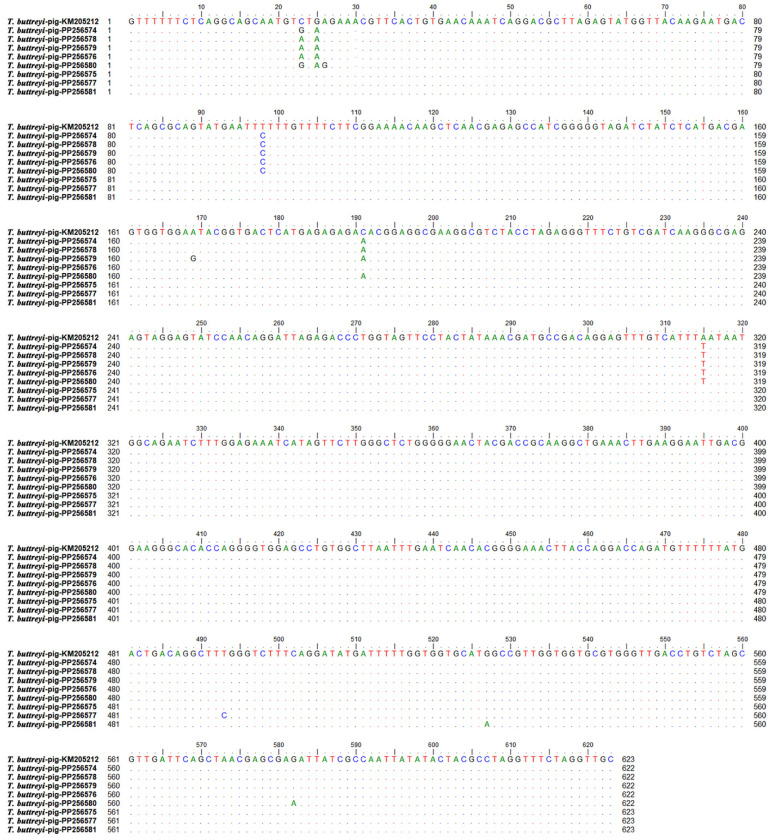
Alignment of the SSU rRNA sequences of *T. buttreyi* isolates obtained in the present study (PP256574–PP256581) and that of a *T. buttreyi* isolate from a previous study (KM205212). Dots represent nucleotides identical to the consensus sequence, while dashes indicate base deletion.

*T. buttreyi*-positive samples were sequenced, and eight distinct sequence types exhibiting 98.56%–99.84% sequence identity were obtained (Figure 2). These sequences are publicly available at GenBank (accession numbers PP256574–PP256581). One sequence (PP256575) showed 100% identity with those of *T. buttreyi* from pigs (KX833156 and KM205212) and cattle (MK880285) from different areas of China, as well as pigs from the Philippines (JX565058) and Germany (MK801506), and horses (AY886859) from the Czech Republic. One sequence (PP256577) was identical to those of *T. buttreyi* from wild boars (AY886865) and Hanuman langur (HQ149978) from the Czech Republic, as well as pigs (JX565053) from the Philippines. Of the remaining six sequences, each was nearly identical to one of the sequences derived from pigs (KX833155, KX833156, and KX833158) in Anhui Province, China.

**Figure 3:**
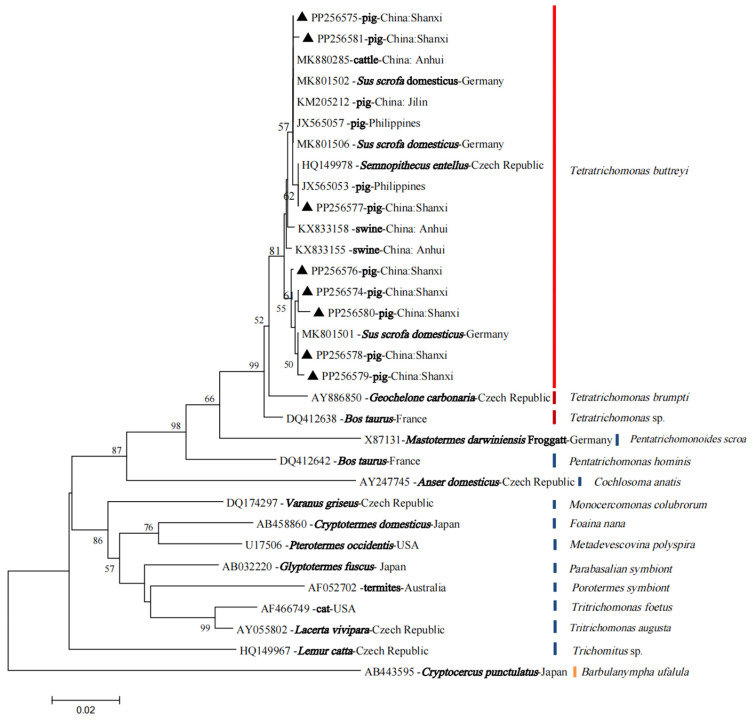
Phylogenetic relationships among trichomonad isolates inferred with a neighbor-joining analysis based on the nucleotide sequences of the SSU rRNA gene, rooted using *Barbulanympha ufalula* as an outgroup. The SSU rRNA sequences generated in the present study are indicated by black triangles.

Though some SSU rRNA sequences obtained in the present study (such as PP256574 and PP256576) did not show 100% identity with the reference sequences in GenBank, the phylogenetic tree confirmed without doubt that all these sequences belonged to *T. buttreyi* based on the NJ method (Figure 3).

## 4. Discussion

Of the techniques developed for the diagnosis of *T. foetus* infection (fecal culture, direct fecal smear examination, and single-tube nested PCR), the single-tube nested PCR showed higher sensitivity and specificity [19]. Meanwhile, a previous study using nested PCR amplification of a fragment of the SSU rRNA gene successfully distinguished and identified the trichomonads isolated from pigs to species level [10]. Hence, we molecularly examined trichomonads in pig fecal samples in Shanxi Province in the present study. The results obtained represent the first molecular evidence for the existence of *T. foetus* and *T. buttreyi* infections in pigs in Shanxi Province, North China.

The prevalence of trichomonads detected in this study (50.00%) was higher than that reported for pigs in two Chinese provinces, Jilin (43.04%) and Anhui (47.40%) [10,16]. Meanwhile, the infection rate of *T. foetus* in pigs observed in the present study (0.28%) was lower than that reported for pigs in other parts of the world, such as Poland (16.28%), Australia (64.50%), and Japan (56.25%) [20,21,22]. Moreover, a statistically significant difference in the prevalence of *T. buttreyi* among age groups was observed in the present study, and pigs aged > 6 months had a 2.26 times higher risk of infection compared with those aged ≤4 months. In addition, the prevalence of *T. buttreyi* varied in different counties. The differences in the prevalence may result from various variables, such as the examination method used, sample size, seasonality, and geographical region.

A previous study showed that the prevalence of *P. hominis*, *T. buttreyi*, and *T. foetus* in pigs in Jilin Province, Northeast China, was 24.05%, 14.57%, and 12.03%, respectively [10]. However, all pig fecal samples were tested negative for *P. hominis* in this study. Notably, our findings showed that *T. buttreyi* was the most common species, and only one sample was tested positive for *T. foetus*. The results were similar to those of a previous study in Anhui Province, in which *T. buttreyi* was detected as a predominant species, and a low prevalence of *T. foetus* was observed [16].

Two sequences obtained in the present study showed 100% identity with those of *T. buttreyi* isolates deposited previously in GenBank, regardless of the geographical locations, suggesting the presence of strong clonality among them. In addition, minor allelic variations were also present in the SSU rRNA sequences from the *T. buttreyi* isolates obtained in this study. The results were similar to those obtained in the previous studies [10,23], which mentioned that the minor genetic distinctness might be due to the expected variation within the multiple copies of the SSU rRNA genes in any given genome or due to adaptation to different hosts and surrounding environments.

## 5. Conclusions

The present study revealed a low prevalence of *T. foetus* but extensive *T. buttreyi* infection in pigs in Shanxi Province, North China. Region and age were found to be significantly associated with *T. buttreyi* infection. For *T. buttreyi*, two representative sequences displayed 100% homology with the reference sequences available in GenBank. In addition, one *T. foetus* isolate and some *T. buttreyi* isolates showed minor allelic variations compared with those reported previously. These findings not only enrich the knowledge of the distribution of these protozoon species in pigs in China but also have implications for developing effective control strategies.

## Figures and Tables

**Table 1:** The prevalence of *Tetratrichomonas buttreyi* in pigs in Shanxi Province, North China.

Factor	Category (County)	No. Tested	No. Positive	Prevalence % (95% CI)	OR (95% CI)	*p*-Value
Region	Qi	68	55	80.88 (71.54–90.23)	10.92 (5.25–22.72)	<0.001
	Shanyin	111	31	27.93 (19.58–36.27)	Reference	
	Jishan	183	94	51.37 (44.12–58.61)	2.73 (1.64–4.52)	
Age	Month ≤ 4	98	36	36.73 (27.19–46.28)	Reference	0.008
	4 < Month ≤ 6	139	73	52.52 (44.22–60.82)	1.91 (1.12–3.23)	
	Month > 6	125	71	56.80 (48.12–65.48)	2.26 (1.32–3.89)	
Total		362	180	49.72 (44.57–54.87)		

## Data Availability

The data sets supporting the results of this article have been submitted to GenBank and the accession numbers are shown in the article.
